# Effect of Particle Sizes of Nickel Powder on Thermal Conductivity of Epoxy Resin-Based Composites under Magnetic Alignment

**DOI:** 10.3390/polym11121990

**Published:** 2019-12-02

**Authors:** Zheng Jin, Fei Liang, Wenzhong Lu, Jinhang Dai, Shunliang Meng, Zihang Lin

**Affiliations:** School of Optics and Electronic Information, Huazhong University of Science and Technology, Wuhan 430074, China; M201772240@mail.hust.edu.cn (Z.J.); lwz@hust.edu.cn (W.L.); M201872156@mail.hust.edu.cn (J.D.); M201972286@mail.hust.edu.cn (S.M.); M201972400@mail.hust.edu.cn (Z.L.)

**Keywords:** polymer-matrix composites (PMCs), anisotropy, thermal properties, analytical modelling

## Abstract

Magnetically oriented three-phase composite systems of epoxy resin, aluminum nitride, and nickel have been prepared, the thermal conductivity of composites filled with nickel powder with different particle sizes and content under different applied magnetic fields was studied. The vibrating scanning magnetometer (VSM) and scanning electron microscopy (SEM) were applied to investigate the dispersion of nickel powder in the composites. The results showed that the anisotropic thermal conductivity of the composites treated by applied magnetic field forming chain structure is obtained. The epoxy resin-based composites filled with 30 vol% aluminum nitride with particle size of 1 μm and 2 vol% nickel powder with particle size of 1 μm and aligned with vertical magnetic field have the highest thermal conductivity (1.474 W/mk), which increases the thermal conductivity of the composites by 737% and 58% compared to the pure epoxy resin (0.2 W/mk) and the composites filled with 30 vol% aluminum nitride (0.933 W/mk). In addition, we simulated the influence of nickel powder particles with different particle sizes and arrangements on the thermal conductivity of the composite material in COMSOL Multiphysics software, and the results were consistent with the experimental results.

## 1. Introduction

With the rapid development of the electronic industry towards miniaturization and high integration, thermal management of electronic devices has become an increasingly important issue [1,2,3,4]. Poor heat dissipation will lead to the failure of devices to work normally, and even permanent damage to components [5,6,7]. Polymers have excellent mechanical properties and poor thermal conductivity [8,9], to increase the thermal conductivity of composites, the dispersion in polymers matrix of high thermal conductivity solid filler materials such as carbonaceous materials (carbon nanotubes, graphene) [10,11,12], metals (silver, copper) [13,14,15], and ceramics (AlN, BN, Al_2_O_3_) [16,17,18] has been studied. In general, when using conventional methods to prepare composite materials, a very high filling amount (usually more than 50% by volume) is required to achieve high thermal conductivity. However, the mechanical properties of composites with high packing loads are usually poor. This requires composites at a low packing concentration have a high thermal conductivity [19]. the common methods that can improve the thermal conductivity of materials under the condition of low content of fillers are as follows: 1) fill the composite materials with mixed fillers; 2) align the filler with the heat flow.

Filling composites with mixed fillers is always an effective method to improve the thermal conductivity and to reduce the overall particle filling amount. Yao, W.C. et al. [4] prepared the epoxy resin composites filled with 22.5 wt% dopamine modified micro-BN and 7.5 wt% KH550 modified nano-Al_2_O_3_, the composites presented a high thermal conductivity of 1.182 W/mK, which was about 700% higher than neat epoxy resin. Teng, C.C. et al. [20] prepared composites filled with 25% AlN and 1% multiwalled carbon nanotubes (MWCNTs), the thermal conductivity of it reached 1.21 W/mK, which is the thermal conductivity of epoxy-containing 50 vol% pristine AlN (1.25 W/mK). However, these mixed fillers are still randomly distributed in the composite material. If they can be arranged in a directional way, the thermal conductivity of which may continue to increase.

The use of magnetic fields during the process of the preparation of polymer composite materials can be also an effective way to orientat the filler [21,22,23]. This situation allows increasing the anisotropy of the composite and therefore preferential directions for the heat transport are created along the aligning direction of the filler aggregate. Li Bin et al. prepared composites containing oriented graphene and carbon nanotubes that were aligned under a 10 T strong magnetic field [6]. The thermal conductivity of composite filled with 0.6 wt% MWCNTs of anisotropy reaches about 0.279 W/mK, which is 57% higher than that of PDMS (0.178 W/mK). the thermal conductivity of 10 T-treated anisotropy composites with 3 wt% GNPs content (0.488 W/mK) shows an increase of 49% and 89% compared to composites filled with random (0.327 W/mK) and anisotropy ⊥ samples (0.258 W/mK), respectively. Kiho Kim et al. prepared composites filled with AlN particles coated with Fe_3_O_4_ and aligned in a magnetic field, the thermal conductivity of it can reach 3.15 W/Mk, which shows an enhancement of 85%, compared to non-magnetically treated composites (1.70 W/mK) [24].

In our work, we combined the two methods mentioned above, AlN and Ni were directly mixed as fillers and aligned under the magnetic field provided by the permanent magnet, aiming at investigating the effects of magnetic field-aligned nickel powders of different particle sizes on the thermal conductivity of composite materials. In addition, we established models in COMSOL to simulate the contribution of nickel powder of different particle sizes arranged in different directions under the induction of the magnetic field to the thermal conductivity of the composite material to verify the law in the experiment.

## 2. Experimental

### 2.1. Materials

Epoxy (EP) monomers of diglycidyl ether of bisphenol-F (DGEBF, YDF-170) were supplied by Nanya Technology Corporation (Nanya, China). The curing agent of 2-ethyl-4-methylimidazole (EMI-2,4, AR), and HCCP (97%) were supported by Shanghai Aladdin Biochemical Technology Co., Ltd (Shanghai, China). Spherical aluminum nitride powders with a mean diameter of 1 μm (1 μm-AlN) were purchased by Toyo Aluminum Co., Ltd (Osaka, Japan). Spherical Nano-Ni powder with a mean diameter of 50 nm (50 nm-Ni) was purchased from Beijing deco island gold technology co., LTD (Beijing, China). Ni powder having an average particle size of 1 μm (1 μm-Ni) and 10 μm (10 μm-Ni) were purchased by Qinghe Huiguang metal materials co. LTD (Xingtai, China).

### 2.2. Preparation of AlN/Ni/ Epoxy Resin Composites

**Figure 1:**
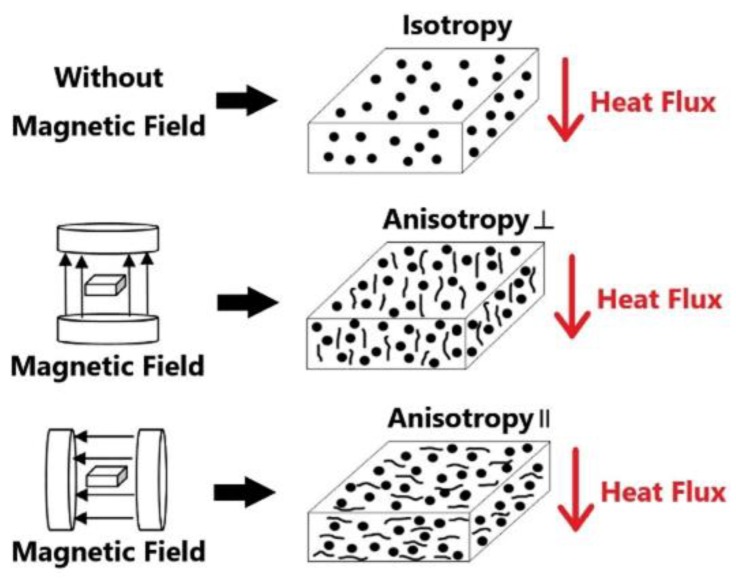
Magnetic treatments applied to the samples during curing.

AlN/Ni/EP composites were fabricated using the following procedure. Typically, stoichiometric AlN, nickel powders, EP monomers, and 6 wt% (relative to EP resin) curing agent EMI-2,4 were blended by using a mechanical stirrer at 50 °C for 1 h, and then under vacuum at 60 °C for 20 min. The obtained mixtures were poured into a mold and cured according to programmed-temperatures of 60 °C for 2 h and 150 °C for 5 h. For all composite samples used in the magnetic alignment experiments in this work, the volume fraction of AlN was 30 vol%. Figure 1 shows the different magnetic treatment methods of samples during curing. When three different types of samples are placed horizontally, the direction of thermal conductivity measurement is always along the vertical direction. After the sample is treated by the vertical magnetic field, the sample is named as anisotropy ⊥ direction, and the sample is named anisotropy ∥ direction after the sample is treated by the horizontal magnetic field.

### 2.3. Characterization

The morphologies of the samples were investigated by scanning electron microscopy (Zeiss Gemini SEM; Ostalbkreis, German). The sample of composite was placed into liquid nitrogen and then fractured along the alignment direction. The dispersion and orientation of the hybrid fillers in polymer composites were observed using scanning electron microscopy (SEM) and energy dispersive spectroscopy (EDS). The density ($\rho_{m}$) of the polymer composites was measured by using the Archimedes method. The relative density ($\rho_{r}$) of the polymer composites was calculated by using Equation.

Differential scanning calorimetry (DSC) was performed on a DSC 200PC (Netzsh Crop., Selb, Germany) instrument to obtain specific heat capacity ($C_{p}$). The thermal diffusivity (a) of the polymer composites was measured by using the laser flash method (LFA-467 Nanoflash apparatus, NETSZCH, Selb, Germany), with the laser flash pulse directed through a specimen at room temperature. The thermal conductivity (k) of the polymer composite was obtained from the results of density, thermal diffusivity and specific heat capacity measurements calculated with Equation.

$$k={\alpha \rho }_{m}C_{p}$$

Magnetic properties were measured on a vibrating scanning magnetometer (VSM, Lake shore, 7404 series VSM, Columbus, OH, USA).

There is a rectangular model, assume that the heat flow through the model at z = 0 is *q*_H_. The temperature of the entire model (*T*_0_) was 293 K at time *t* = 0. The temperature profiles of the model at different times (*t* = *τ*_1_ and *t* = *τ*_2_) were simulated. The change in temperature relative to *T*_0_ at *z* = *z* and *t* = *τ* can be expressed using the following functions [25]:

(1) $$\theta \left(z,t\right)=\frac{2q_{H}\sqrt{\alpha \tau }ierfc\left(\eta \right)}{\lambda }=T-T_{0}$$

(2) $$\eta =\frac{z}{2\sqrt{\alpha t}}$$

(3) $$q_{H}=-\lambda {\frac{\partial T}{\partial z}|}_{z=0},$$

where *λ* is the thermal conductivity of the material, *α* is the thermal diffusion coefficient of the material, *ierfc*(*η*) is the first integral of the complementary error function. From the simulation data results, θ(0,*τ*_1_) and θ(1,*τ*_2_) can be obtained as follows:

(4) $$\theta \left(0,\tau_{1}\right)=1.1282\frac{q_{H}}{\lambda }\sqrt{\alpha \tau_{1}}$$

(5) $$\theta \left(l,\tau_{2}\right)=\frac{2q_{H}}{\lambda }\sqrt{\alpha \tau_{2}}ierfc\left(\eta_{0}\right)$$

(6) $$\eta_{0}=\frac{l}{2\sqrt{\alpha \tau_{2}}}.$$

By dividing Equation (5) with Equation (4), we obtain Equation (7) as follows:

(7) $$ierfc\left(\eta_{0}\right)=0.5642\sqrt{\frac{\tau_{1}}{\tau_{2}}}\frac{\theta \left(l,\tau_{2}\right)}{\theta \left(0,\tau_{1}\right)}.$$

The values on the right side of Equation (7) can be obtained from the simulation results.

From Equation (6), *α* can be obtained using Equation (8):

(8) $$\alpha =\frac{1}{4\tau_{1}}\frac{l^{2}}{\eta_{0}{}^{2}}.$$

Then, *λ* can then be obtained from Equation (9):

(9) $$\lambda =1.1282\frac{q_{H}}{\theta \left(0,\tau_{1}\right)}\sqrt{\alpha \tau_{1}}.$$

## 3. Results and Discussion

### 3.1. Structural Analysis

**Figure 2:**
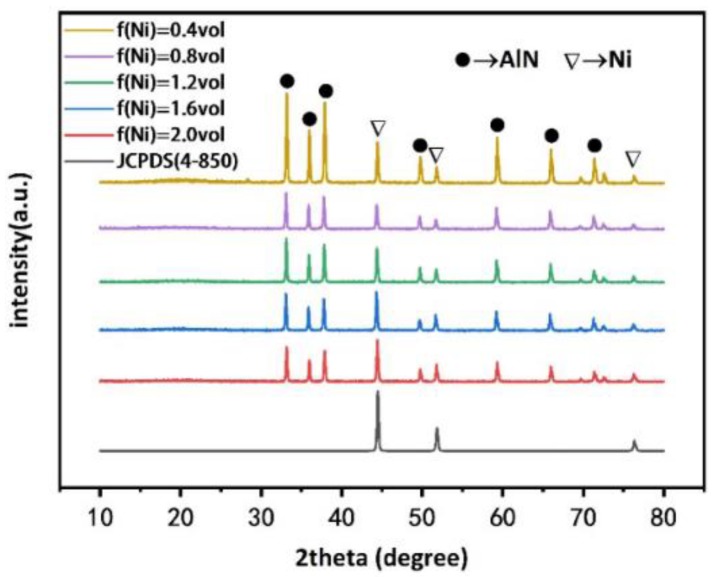
XRD patterns of composites containing AlN (30 vol%) and nickel powder at varying volume fractions.

Figure 2 shows the XRD result of Composites filled with nickel powder of different volume fractions. The characteristic diffraction peaks of AlN (2θ = 33.2°, 36.0°, 37.9°, 49.8°, 59.3°, 66.0°, 69.4°, 71.2° and 72.3°) were assigned to the (100), (002), (101), (102), (110), (103), (200), (112), and (201) reflections, respectively [26]. the characteristic peaks (2θ = 44.4°, 51.6° and 76.1°) are assigned to the (111), (200) and (220) reflections, respectively. These are characteristic of nickel, in accordance with the JCPDS (4-850) [27]. Since epoxy resin does not have crystal structure, the diffraction peak of epoxy resin cannot be detected by XRD.

### 3.2. Morphology

**Figure 3:**
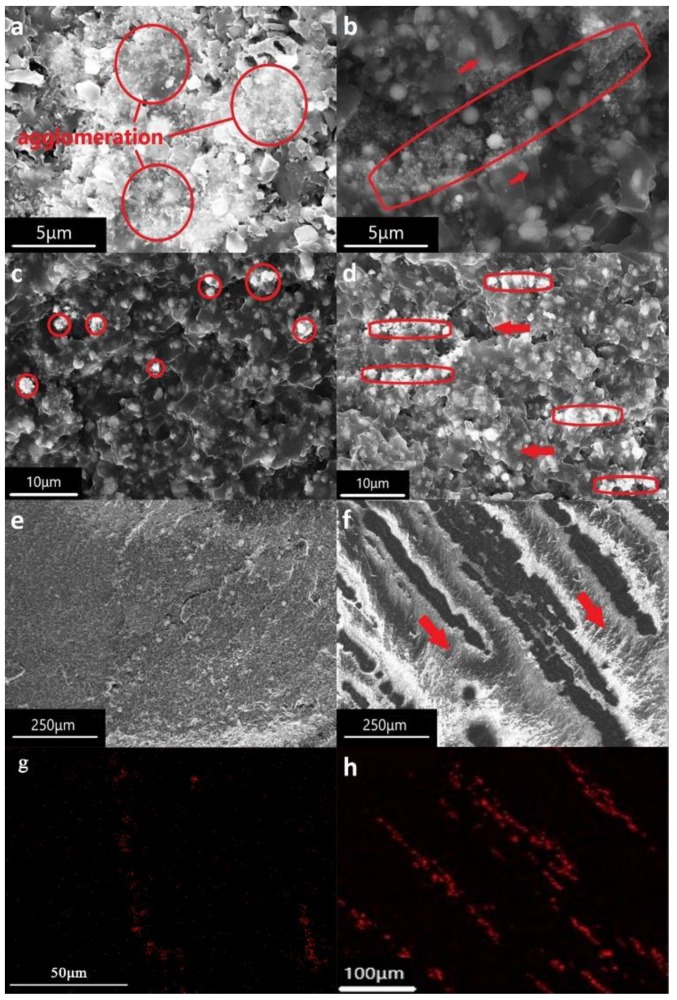
Scanning electron microscopy (SEM) images of the fracture surface of (**a**) randomly distributed composites containing 50 nm-Ni; (**b**) magnetically aligned composites containing 50 nm-Ni; (**c**) randomly distributed composites containing 1 μm-Ni; (**d**) magnetically aligned composites containing 1 μm-Ni; (**e**) randomly distributed composites containing 10 μm-Ni; (**f**) magnetically aligned composites containing 10 μm-Ni; energy dispersive spectroscopy (EDS) of composites filled with (**g**) 1 μm-Ni; (**h**) 10 μm-Ni.

During the curing process, spherical particles of nickel in the composites of liquid state are oriented under the alignment of the magnetic field. In order to get a more accurate structural analysis of the distribution of nickel with or without magnetic alignment in the composite material, the morphology and microstructure was examined by SEM and EDS. In preparing the pictures, samples are all the composites containing 30 vol% AlN and 1.6 vol% nickel. They were placed into liquid nitrogen and fractured along the alignment direction. The red arrows in the figures represent the direction of the applied magnetic field. Figure 3a shows that nano-Ni powders that agglomerated to micron-scale are randomly distributed in the composites, note that the small particles circled in red are nickel powder and the larger particles not circled are aluminum nitride. Figure 3b shows that nano-Ni powder similarly aggregates into micron-sized particles which are aligned with the magnetic field, however, due to the large surface forces of nanoparticles, it can be found that even under the induction of magnetic field, nanoparticles are still not directly connected to form a continuous nickel powder chain, and the spacing between particles is still large. Figure 3c shows 1 μm-Ni which is circled in red randomly distributed in the composites. Because the conductivity of nickel is stronger than that of AlN, the shining particle in SEM is Ni, the non-shining particle is AlN. Figure 3d presents the microstructure of epoxy resin filled with AlN and 1 μm-Ni powder aligned with the magnetic field. The images show the alignment of Ni powder with the direction of the magnetic field. The nickel powder direct contact with each other, forming a continuous nickel powder chain, which is obviously different from the morphology of samples filled with nanometer nickel powder showed in Figure 3b. Figure 3e,f show the fracture surface of samples filled with 10 μm nickel powder without and with magnetic field alignment. Comparing these two pictures, it is found that the continuous particle chains are created by applying a magnetic field. It indicates that nickel is indeed oriented along the magnetic field in the composites and forms a local continuous nickel particle chain, which plays a significant role in accelerating the conduction of heat flow. Figure 3g,h show the image of Ni elemental map collected from a sample filled with 1 μm-Ni and 10 μm-Ni. These can clearly prove that 1 μm-Ni and 10 μm-Ni powder form many continuous Ni powder chains in the composite material under the alignment of the magnetic field.

### 3.3. Magnetization Study

**Figure 4:**
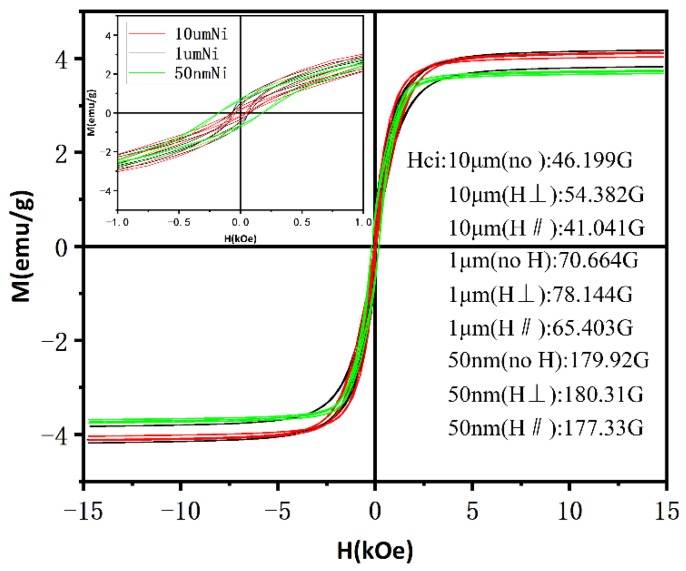
Magnetization curves of composite material containing nickel powder of different particle sizes.

The magnetic properties of the composites containing nickel powder with different magnetic fields were characterized by a vibrating scanning magnetometer (VSM). The samples are all the composites containing 30 vol% AlN and 1.6 vol% nickel. Figure 4 is the result of the magnetization test of samples at 298 K, the upper left corner figure is the result when −1 kOe < H < 1 kOe. Firstly, it shows that the coercive force of the composites filled with 50 nm-Ni is greater than that filled with 1 μm-Ni, which is greater than the composites filled with 10 μm-Ni. The value that represents the critical diameter (DC) for multidomain/monodomain behavior is about 40 nm [28], it means ferromagnetic powder with particle sizes above 40 nm are multidomain. In the multidomain range, above DC, and the coercivity decreases as the particle size increases. The coercivity of 50 nm nickel powder is up to 180 gausses, which is 4.5 times that of the 10 μm-Ni sample and 2.6 times that of the 1 μm-Ni sample. Compare these with the previous SEM image. It is not difficult to find that the smaller the particle size is, the more difficult it is to form a continuous nickel powder chain under the same applied magnetic field. Secondly, it shows that among the samples filled with nickel powder of the same particle size, the coercive force of the composites aligned by a vertical magnetic field (H⊥) is greater than that without magnetic alignment, which is greater than that aligned by a horizontal magnetic field (H∥). Note that the direction of the magnetic field during the VSM test is the same as the direction of H∥, while the situation that the coercive force of the composites aligned by H∥ is not equal to that of the composites aligned by H⊥ illustrates the anisotropic structure of the Ni powder in the composites.

### 3.4. Thermal Conductivity

**Figure 5:**
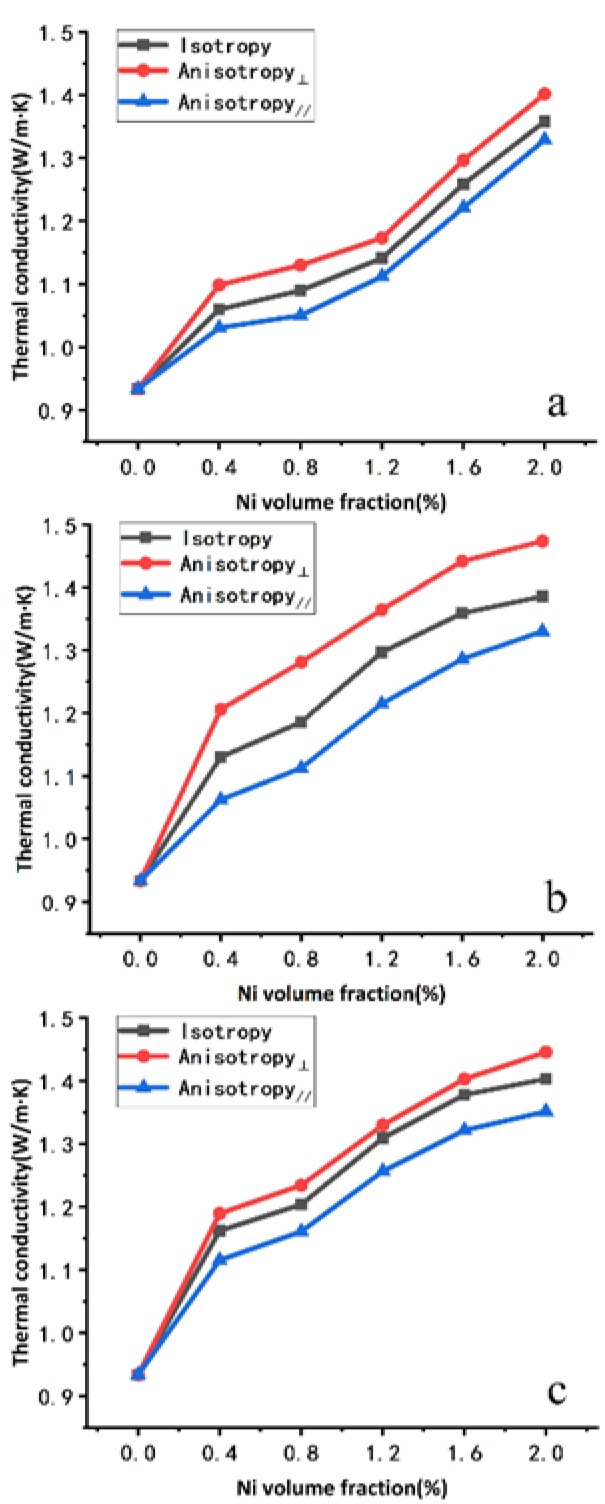
Thermal conductivity of the composites filled with (**a**) 50 nm-Ni, (**b**) 1 μm-Ni and (**c**) 10 μm-Ni at varying filler contents and subjected to different magnetic field treatments.

The thermal conductivity of the composites that filled with AlN and nickel powder of different particle sizes and contents under different applied magnetic fields are presented in Figure 5a–c. The effect of the contents and particle size of nickel powder on the thermal conductivity of composites is also presented for comparison, the thermal conductivity data of different series composites were obtained: The magnetic field is perpendicular to the horizontal plane (⊥); The magnetic field is parallel to the horizontal plane (∥); and a random distribution of nickel particles in the composites. Note that the heat flow direction is perpendicular to the ground when testing the thermal conductivity of composites.

From Figure 5, we can observe a nearly linear increase of the thermal conductivity when Ni powders filler concentration increases. This increasing was foreseeable because nickel powders have a higher thermal conductivity than epoxy resin. In addition, for a given content, the composite filled with nickel powder aligned parallel to the main heat flow exhibits a higher thermal conductivity than the one with a perpendicular or random distribution of nickel powder. According to Figure 5, composites filled with 30 vol% AlN and 2 vol% Ni under the vertical magnetic field have the highest thermal conductivity in each figure. It is because that the magnetic field arranges the Ni particles in chain-like structures, which facilitates the heat conduction through them and therefore enhances the effective longitudinal thermal conductivity of the composite, with respect to their corresponding values when the particles are distributed parallel to the main heat flux direction (⊥). In addition, according to Figure 5, the particle size of Ni powder also has a significant effect on the thermal conductivity of the composite under the external magnetic field. The thermal conductivity of the composites filled with nickel powder with particle sizes of 50 nm, 1 μm, and 10 μm reached 1.401, 1.474 and 1.445 W/mK, respectively. Among them, the thermal conductivity of composites filled with 1 μm-Ni (1.474 W/Mk) shows enhancements of 737% and 58%, compared to that of pure epoxy resin (0.2 W/mK) and that of composites filled with only 30 vol% 1 μm-AlN without nickel powder (0.933 W/mK). Moreover, Figure 5a–c also show that the thermal conductivity of the composites with the same nickel content increases with the increase of the particle size of nickel powder without an applied magnetic field. The main reason is that the smaller the particle size of nickel powder, the larger the surface area, the higher the interfacial thermal resistance between nickel powder and epoxy resin matrix, and the lower the thermal conductivity of the composites. In addition, under the same applied magnetic field and the same content of nickel powder, the change of thermal conductivity of composites with 1 μm-Ni powder is more obvious than that of composites with 10 μm-Ni powder. This is because the smaller the particle size of nickel powder, the more the number of nickel powder chains formed under the same applied magnetic field, the more obvious the change of thermal conductivity of composites. However, under the same applied magnetic field and the same content of nickel powder, the change of thermal conductivity of 50 nm Ni powder composite material is not as obvious as that of 1 μm-Ni powder composite material. This is mainly because it is difficult for nano-nickel powder to form a continuous nickel powder chain under the action of an applied magnetic field. Figure 3b also shows that there is no obvious chain structure of nickel powders in the composite of 50 nm Ni powders under the applied magnetic field. We know that nanoparticles are always easier to agglomerate. The main reason for agglomeration is the van der Waals force between nanoparticles [29], and the large surface area and high surface energy of nanoparticles lead to a stable state of agglomeration [30]. In addition, the high coercivity of the composite filled with 50 nm-Ni makes it more difficult to form a continuous nickel powder chain under the same magnetic field orientation. Therefore, the interfacial thermal resistance and thermal conductivity of the composite filled with 50 nm-Ni did not decrease significantly.

### 3.5. Simulation Verification

**Figure 6:**
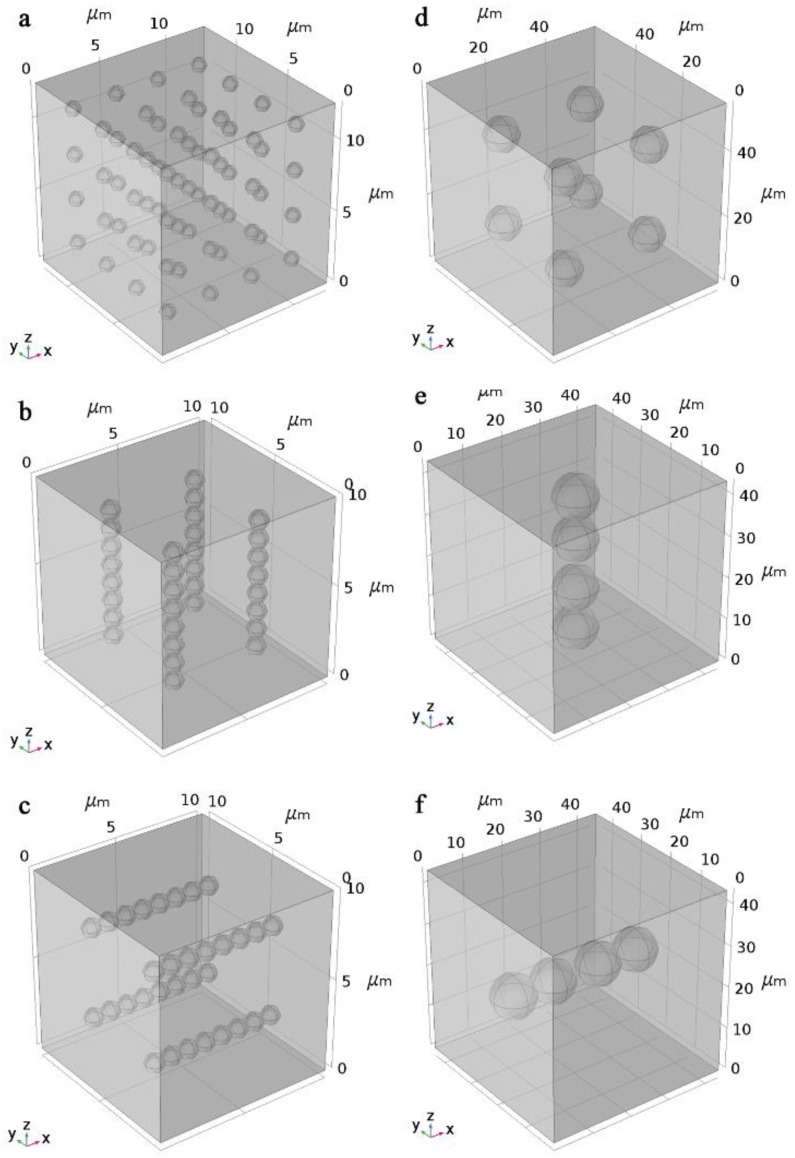
Models of composites containing 1 μm-Ni in the (**a**) absence and presence of (**b**) a vertical or (**c**) horizontal magnetic field. Models of composites containing 10 μm-Ni in the (**d**) absence and presence of (**e**) a vertical or (**f**) horizontal magnetic field.

To verify the effect of particle sizes of nickel powder on thermal conductivity of epoxy resin-based composites under magnetic alignment in Figure 5, the models of sample filled with different particle sizes of Ni were established in COMSOL software (COMSOL Inc., Stockholm, Sweden) [31,32,33]. Because the agglomeration phenomenon of nano-Ni powder is serious, it is difficult to simulate the agglomeration phenomenon in the model. Therefore, in this paper, we only established 1 μm-Ni and 10 μm-Ni filling models but did not establish the nano-Ni powder filling model. The model assumes that epoxy filled with 30 vol% AlN evenly distributed as a uniform medium. The spherical particles in the models are all nickel powder. The volume fraction of nickel powder in the model was all constantly 1.6 vol%. The models shown in Figure 6a, d were established first. Then, the models were repeated in the *z*-direction until the thickness reached the millimeter level. A heat source was added with constant heat flow *q*_H_ at *z* = 0. The temperature of the entire model (*T*_0_) was 293 K at time *t* = 0. The temperature profiles of two objects at different times (*t* = *τ*_1_ and *t* = *τ*_2_) were simulated. The thermal conductivity of the model can be obtained through the Equations listed in the Characterization section.

By adjusting the thermal conductivity of the uniform medium, the total thermal conductivity of the material filled with 1 μm-Ni and 10 μm-Ni reached 1.35 W/mK and 1.378 W/mK, respectively, as experimentally observed in Figure 5.

In subsequent simulation experiments, the thermal conductivity of the well-adjusted uniform medium remained unchanged and the equivalent models filled with 1 μm-Ni or 10 μm-Ni powder in different chain-like structure arrangements as shown in Figure 6b–f were established. To clearly observe the difference in diffusion distance of the isothermal surface between different models, the numbers of models were increased until the thickness of the model in the *z*-direction reached 200 μm. The temperature of the entire model was initially set as 293 K, and the surface at 0 μm was set as a heat source of 400 K. The diffusion distances of the isothermal surface of different models at 20 ms were observed. The diffusion distances of isothermal surfaces of the models with different structures at the same time are different, and the relationship between the thermal conductivity of the models can be qualitatively determined based on the diffusion distance.

**Figure 7:**
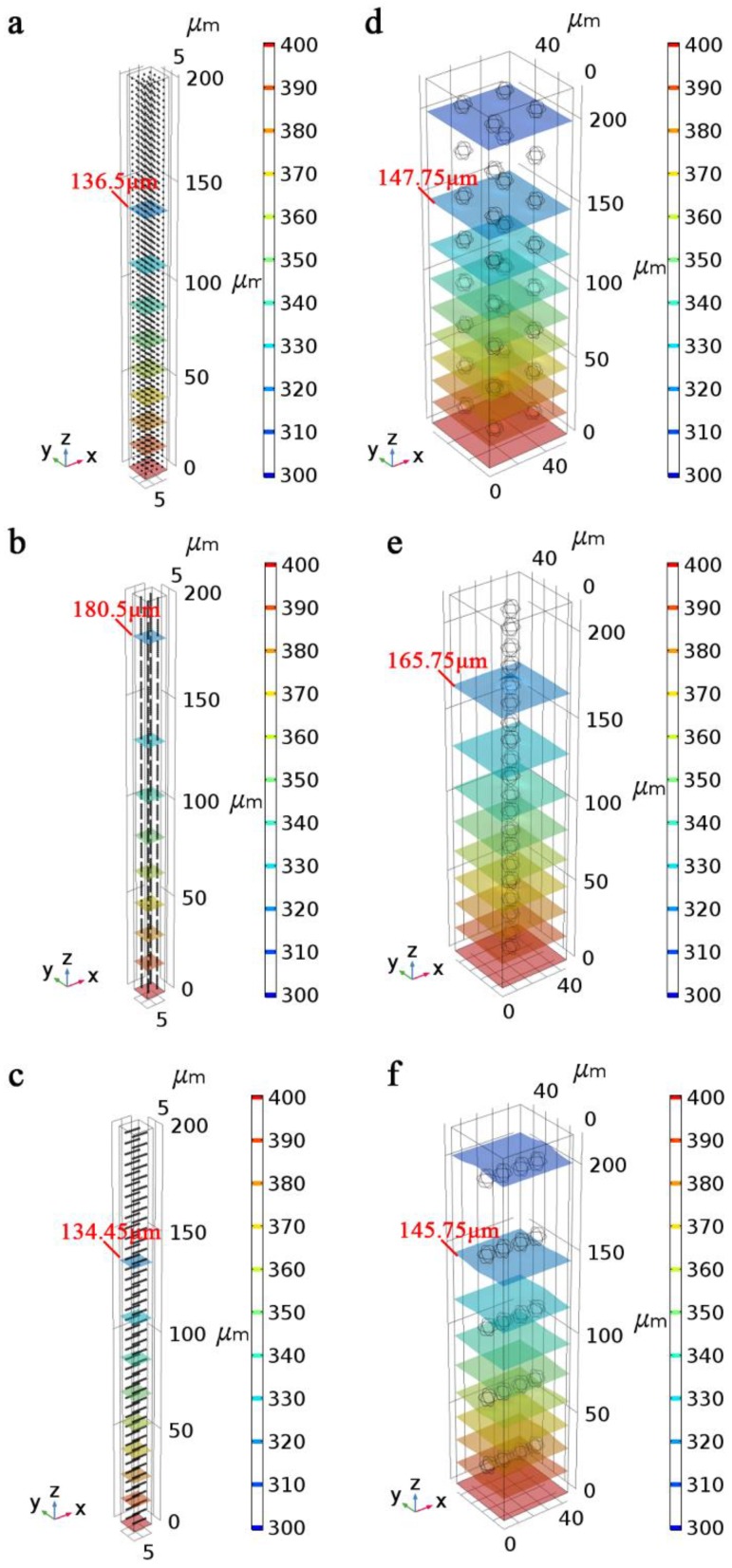
Simulation results of the composite models containing 1 μm-Ni in the (**a**) absence and presence of (**b**) a vertical or (**c**) horizontal magnetic field. Simulation results of the composite models containing 10 μm-Ni in the (**d**) absence and presence of (**e**) a vertical or (**f**) horizontal magnetic field.

The simulation results, presented in Figure 7, reveal that the thermal conductivity of 1 μm-Ni model with random distribution is lower than that of 10 μm-Ni model with random distribution, but that of 1 μm-Ni model with vertical arrangement is higher than that of 10 μm-Ni model with vertical arrangement. This phenomenon is consistent with changes in the thermal conductivity observed between composites with 1 μm-Ni powder and 10 μm-Ni powder shown in Figure 5 in the presence or absence of an applied magnetic field. In addition, for the models with the same particle size, the heat transfer rate of the model with vertical distribution of nickel powder was the highest, whereas the heat transfer rate of the model with horizontal distribution of nickel powder was the lowest; these results confirmed the anisotropic thermal conductivity observed for samples under magnetic field alignment.

## 4. Conclusions

The effect of the particle size of nickel powder on the thermal conductivity of composite systems consisting of epoxy resin, aluminum nitride, and nickel under different magnetic alignment was studied. Vibrating scanning magnetometry results showed that when the particle size of Ni powder was larger than the critical diameter, the larger the particle size of Ni powder, the smaller the coercivity of the composite material. When the applied magnetic field was parallel (in contrast to being perpendicular) to the magnetic field direction of the coercivity test, the smaller the coercivity of the composite material. The SEM results revealed that the formation of a continuous chain structure did not occur in the composite with nano-Ni powder subjected to an applied magnetic field. In contrast, a continuous chain structure was observed in the composites with 1 μm and 10 μm Ni powder subjected to an applied magnetic field. The thermal conductivity results showed that the smaller the particle size of the micrometer Ni powder, the greater the number of Ni powder chains formed in the composite under an applied magnetic field, and the greater the change of thermal conductivity of the corresponding composite. However, under the same applied magnetic field and the same content of nickel powder, changes in the thermal conductivity of 50 nm Ni powder composite material were not as obvious as changes observed for the 1 μm Ni powder composite material. The simulation results confirmed these findings and the anisotropic thermal conductivity of the composites in the presence of a magnetic field.
